# The value of real-time continuous glucose monitoring in premature infants of diabetic mothers

**DOI:** 10.1371/journal.pone.0186486

**Published:** 2017-10-16

**Authors:** Hean-Pat Saw, Nai-Wei Yao, Cheng-Di Chiu, Jia-Yuh Chen

**Affiliations:** 1 Institute of Medicine, Chung Shan Medical University, Taichung, Taiwan; 2 Chung Kang Branch, Cheng Ching General Hospital, Taichung, Taiwan; 3 School of Medicine, China Medical University, Taichung, Taiwan; 4 Stroke Center, China Medical University Hospital, Taichung, Taiwan; 5 Graduate Institute of Biomedical Sciences, China Medical University, Taichung, Taiwan; 6 Department of Neurosurgery, China Medical University Hospital, Taichung, Taiwan; 7 Department of Pediatrics, Chung Shan Medical University Hospital, Taichung, Taiwan; Centre Hospitalier Universitaire Vaudois, FRANCE

## Abstract

To determine the feasibility of using a real-time continuous glucose monitoring system (RTGMS) in intensive care units, our study focus on preterm infants with diabetic mothers owing to their high risk of blood sugar abnormalities. Thirty preterm babies (*M* = 15 and *F* = 15; ≤ 36 week gestation age) were studied from within 72 hours of delivery. These babies were admitted to the newborn intensive care and were further categorized into groups based on whether their mothers with or without diabetic mellitus. Blood sugar levels were monitored by both RTGMS and the traditional intermittent arterial line (A-Line) glucose method. Continuous glucose monitoring were well tolerated in 30 infants. There were good consistency between RTGMS and A-Line glucose concentration measurements. Of the preterm infants, 33.33% experienced abnormal glucose levels (hypoglycemia or hyperglycemia) between the checkpoint intervals of the intermittent A-Line blood sugar measurements. RTGM showed advantages with regards to reduced pain, greater comfort, the provision of real-time information, high sensitivity (94.59%) and specificity (97.87%) in discovering abnormalities of blood sugar, which are especially valuable for premature infants of diabetic mothers. RTGMS is comparable to A-line measurement for identifying fluctuations in blood glucose in premature infants. RTGMS detects more episodes of abnormal glucose concentration than intermittent A-line blood glucose measurement. High risk infants, especially premature infants with diabetic mothers, should receive more intensive blood sugar level checks by using continuous RTGMS.

## Introduction

Blood glucose level fluctuations after birth are common in high-risk infants. Hypoglycemia is frequently associated with various neonatal conditions such as prematurity, intrauterine growth restriction, and maternal diabetes mellitus (DM). Therefore, screening for hypoglycemia in high-risk situations is recommended [1]. Moreover, excessive intravenous glucose infusion, prematurity, shorter gestation age, small-for-gestational-age birth, use of an inotropic agent, lipid infusions, neonatal diabetes, and sepsis are risk factors for hyperglycemia in infants [2–5].

Infants with hypoglycemia may present with jittering, irritability, lethargy, seizure, or even impaired neurodevelopment [6]. Although most symptoms may be not obvious [3], there is no concrete evidence demonstrating causality between a particular level or duration of hypoglycemia and adverse long-term outcomes [7–9]. The normal range of blood glucose is variable and depends on factors such as birth weight, gestational age, body stores, and feeding status, as well as availability of energy sources and presence or absence of disease. Therefore, the definition of hypoglycemia should instead be flexible and encompass the full scope of the aforementioned considerations. However, for hyperglycemia, studies have reported that it increases the risk of intraventricular hemorrhage, morbidity, mortality [1, 3, 10], and eye disorders [10–12].

Thus, aggressive repeated measurement, screening, and treatment of blood glucose levels in at-risk infants is recommended [13], especially for the premature infants of diabetic mothers because these infants are likely to have hypoglycemic periods shortly after birth and during the first few days of life. All infants of diabetic mothers should be tested for hypoglycemia, even if they have no symptoms, and testing should be continued until the infant's blood glucose remains stable. However, the conventional method of checking blood glucose levels, which occurs intermittently with three to four blood glucose measurements per day, could lead to increased risk of infection, anemia, and episodes of undetected hypoglycemia or hyperglycemia. Furthermore, the duration and severity of blood glucose abnormalities may be ignored.

Several studies conducted in adult intensive care contexts have indicated that outcomes can be improved as a result of either tight control of glucose levels or anabolic effects of insulin [12]. Thus, scholars have recommended aggressive screening and treatment of at-risk infants [14]. The potential advantages of RTGMS include detecting undiagnosed episodes of hypoglycemia and hyperglycemia; providing information about the duration and severity of these episodes, frequencies, and fluctuations; allowing the evaluation of the infant’s response to treatment; reducing the pain and disturbance associated with repeated blood sampling [15]; and supplying trend information to identify and prevent unwanted periods of hypoglycemia and hyperglycemia [16]. Two types of blood glucose monitors were considered in the present research, arterial line (A-line) and RTGMS, which differ in much the same ways: 1) An intermittent blood glucose monitor measures discrete glucose levels with high accuracy levels, whereas a continuous monitor provides multiple glucose level measurements with fair accuracy levels; 2) with an intermittent monitor, current blood glucose levels do not predict future glucose levels, whereas this is possible with a continuous monitor; 3) with an intermittent monitor, it is easy to study every measured blood glucose value over most time periods, but with a continuous monitor, too much data are generated to study all data points; and 4) an intermittent blood glucose monitor requires effort to operate, whereas a continuous monitor does not.

In the present study, we investigated the usefulness and reliability of RTGMS in premature infants admitted to the newborn intensive care unit for detecting the incidence of hypoglycemia and hyperglycemia compared with the traditional intermittent A-line method. We evaluated the usefulness and reliability of RTGMS and whether its measurements correlate with intermittent blood glucose tests. Ultimately, our objective was to prevent vulnerable infants from suffering from puncture pain and to provide more accurate data collection for treatment.

## Materials and methods

### Participants

The study participants were 30 infants who were born <37 weeks of gestational age and admitted to the neonatal intensive care unit in Chung Shan Medical University Hospital, Taichung, Taiwan. They were divided into two groups: those with non-DM mothers (n = 15) and those with DM mothers (n = 15). All premature infants in this study were cared for according to the clinical guidelines of the newborn intensive care unit. Early oral feeding were commenced with breast milk in 8–12 meals per day (maximum: 30 mL/kg/day), depending on the clinical stability and individual’s risk factors. If breast milk was not available, they were fed premature infant formula. The parenteral nutrition was increased daily to reach the nutritional recommendations of a total intake of 3.5–4 g/kg/day of proteins and 2.5–3 g/kg/day of lipids, with the average energy intake of 120 kcal/kg/day, as recommended by the American Association of Pediatrics. We excluded premature infants born with any major congenital malformations, including chromosome abnormally, confirmed or suspected sepsis or pneumonia, in a terminal state on admission, umbilical anomaly, skin infection, or conditions that prevented sensor attachment.

### Ethics statement

This study has been approved by the Institutional Review Board of Cheng Ching General Hospital (IRB No: HP110006). Written informed consent was obtained from each parents or guardians of the minors before any study procedures were performed.

### Real-time glucose monitoring system (RTGMS)

The MiniMed® Paradigm Real-Time System Sensor for glucose monitoring (Medtronic Diabetes, Northridge, CA) was used [17] while infants stayed at the neonatal intensive care unit. Briefly, the glucose sensor (MMT-7003) was mounted inside a hollow needle to allow subcutaneous insertion into the proximal lateral thigh. After insertion, the practitioner observed the insertion site for about 5 min before attaching the sensor to the transmitter to watch for bleeding. If bleeding occurred, steady pressure was applied by sterile gauze or a clean cloth for up to 3 min. Interstitial glucose concentration collected by the RTGMS was converted into an electrical signal that was stored every 10 s. The monitor then averaged the signal every 5 min, providing approximately 288 data points of interstitial glucose concentration per day. Blood glucose concentration were monitored continuously and the values were recorded manually every 3 hours from birth.

### Blood sugar monitoring and infant care

Glucose values from arterial blood were measured and analyzed by standard intermittent sampling. A minimum of three samples were drawn during each 24-hr period and used to calibrate the continuous glucose monitor. In addition, further measurements of the infant were taken based on the clinical judgement of the attending physician. All blood glucose concentrations, feeds, and medications for the management of hypoglycemia or hyperglycemia into the continuous glucose monitor were recorded and reported by nursing staff.

Hypoglycemia and hyperglycemia were defined on the basis of levels clinically used to indicate the need for intervention: ≦40 mg/dl and ≧120 mg/dl, respectively.

For the safety consideration, we took more frequent blood glucose monitoring by RTGMS than A-Line sampling. Briefly, blood glucose concentration was measured with A-line sampling every 6 hours and RTGMS every 3 hours for a 72-hour period. In infants whose glucose level measured ≦40 mg/dl on the RTGMS, artery glucose levels were checked at the same time. A bolus of 10% dextrose solution (2 ml/kg) was administered for 10 min and, if necessary, glucose infusion rates were increased (2mg/kg/min). In infants receiving intravenous dextrose, blood glucose concentrations were measured again 30 min after treatment and monitoring continued every 6 hours, then less frequently as clinically indicated. The intervention criteria for hyperglycemia was defined as a blood glucose concentration ≧180 mg/dl. An insulin pump was started after two consecutive hourly blood glucose levels of ≧180 mg/dl at a rate of 0.05 IU/kg/h, and was titrated by capillary measurements to maintain a glycaemia of 120–180 mg/dl. Reduction of glucose infusion was only considered in cases of unmanageable hyperglycemia after insulin administration.

### Statistical analysis

Results obtained from independent experiments are presented as the mean ± standard error of the mean (SEM). Between-groups differences were tested with analysis of variance (ANOVA) followed by Fisher’s post-hoc tests. Student’s two-tailed t-tests were used to determine the differences between any two group means. All statistical analyses were performed using StatView software (SAS Institute, NC, USA). A P value of <0.05 was taken as statistically significant.

## Results

### RTGMS and A-line monitoring

**Fig 1:**
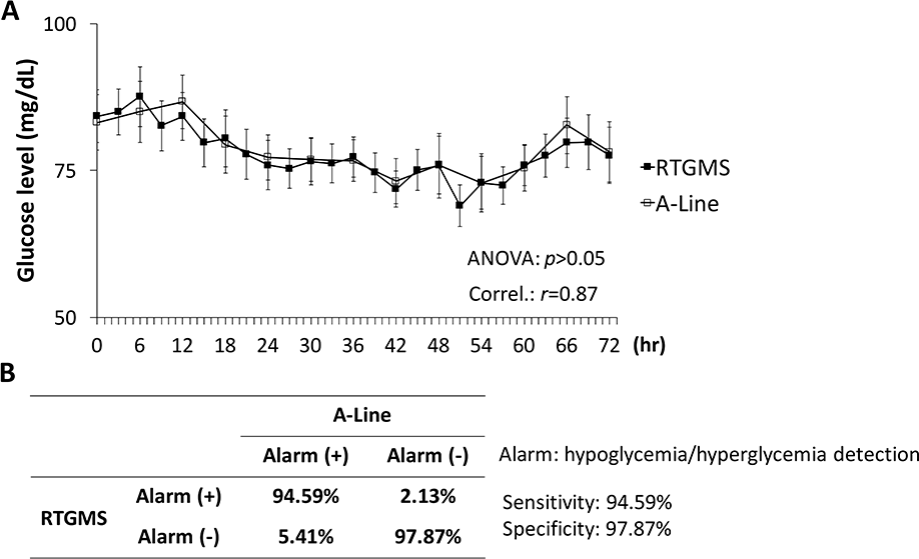
Glucose levels were indicated by A-line and RTGMS throughout the 72-hr study period. (A) Glucose levels of all premature infants were recorded by A-line and RTGMS throughout the 72-hr. (B) The sensitivity and specificity of A-line and RTGMS.

The prevalence of both hypoglycemia and hyperglycemia for each subject was defined as the percentage of studied time spent hypoglycemic or hyperglycemic. The correlation between the RTGMS and A-line measurement methods was determined with Pearson’s correlation. Data are presented as means ± SEM. The results of the analysis showed a strong correlation between the RTGMS and A-line measurements (*r* = 0.87; Fig 1A). Similarly, pairwise t-tests were conducted on the matching time points (the time points at which both RGTMS and A-line measurements were taken). RTGMS and A-line did not differ significantly from one another in terms of identifying hypoglycemia/ hyperglycemia (*p* = 0.54; Fig 1A). In order to estimate the efficacy of RTGMS, sensitivity and specificity analyses were performed (Fig 1B). High sensitivity and specificity were demonstrated for RTGMS (sensitivity: 94.59%, specificity: 97.87%).

### Blood glucose fluctuations in DM and non-DM infants

According to studies from other groups [13, 18, 19], infants with a mother who suffers from diabetes mellitus (DM) exhibit greater fluctuations in blood glucose levels than infants whose mother do not have DM. In the present study, infants were further categorized into one of two groups: infants with DM mothers (DM: *n* = 15) or infants with non-DM mothers (non-DM: *n* = 15). The group details are summarized in Table 1.

**Table 1:** Description of infants comprising the DM and non-DM groups.

	Non-DM mother	DM mother	P value
**Male infants**	n = 7	n = 8	
**Female infants**	n = 8	n = 7	
**Birth weight**	1732.0 ± 87.6	2335.1 ± 261.6	0.022

Data are expressed as percentage and mean±SEM.

**Fig 2:**
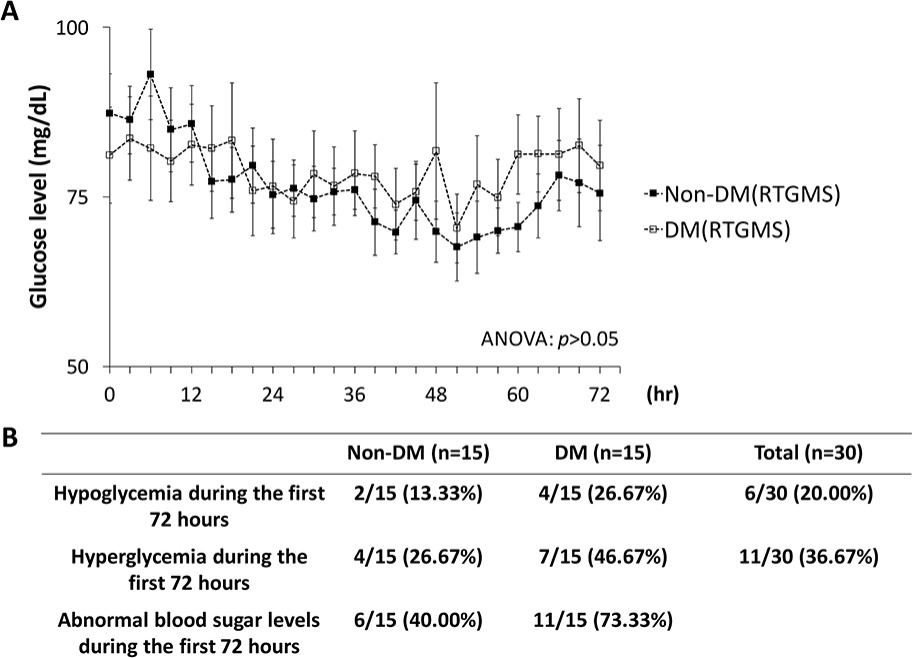
Glucose levels for preterm infants with DM and non-DM mothers. (A) Glucose levels of preterm infants with DM and non-DM mothers were recorded by RTGMS throughout the 72-hr. (B) The proportion (%) of infants with hyperglycemi**a** and hypoglycemia throughout the 72-hr study period was shown.

The two groups were evaluated using a one-way ANOVA comparing the blood glucose measurements taken by RTGMS between the DM and non-DM. As shown in Fig 2A, there was no significant difference between the two groups (*p* = 0.09). Interestingly, the rate of hypoglycemia/ hyperglycemia alarms during the 72-hr study period was numerically higher in DM group than the non-DM group (non-DM: 40.00% vs. DM: 73.33%).

## Discussion

Infants of diabetic mothers are at a high risk of hypoglycemia in the first 24 hours of life. Typically, hypoglycemia occurs within the first few hours after birth, likely caused by persistent hyperinsulinemia in the newborn after the interruption of the intrauterine glucose supply from the mother. Infants of diabetic mothers who are preterm or small for gestational age are at an increased risk of hypoglycemia because glycogen stores are reduced and hyperinsulinemia impairs the ability to mobilize hepatic glycogen [20–22]. In these infants, hypoglycemia may last longer than 2–4 days and may require more prolonged and higher rates of glucose infusion, which, in turn, may increase the possibility of hyperglycemia. Thus, infants of diabetic mothers who are preterm require close blood glucose monitoring for hypoglycemia and hyperglycemia state after delivery and frequently need glucose supplementation, including parental glucose infusion.

In the present study, we demonstrated that RTGMS is a practical method for measuring blood glucose levels over the 72-hours monitoring period and that its noninvasiveness and low complications rate may be superior to the A-line method [23–26]. Our findings support that RTGMS is safe and potentially useful for continuous glucose monitoring, particularly in high-risk infants and premature infants of diabetic mothers.

Further testing should be undertaken to define the cause of persistent hypoglycemia in infants who continue to require glucose infusions at rates exceeding 8–10 mg/kg/min to maintain normal plasma glucose levels beyond the first week of life. Specifically, continuous measurement is helpful for detecting episodes of abnormal blood glucose levels, such as hypoglycemia or hyperglycemia, which traditional intermittent blood glucose methods may miss. In our study, approximately 33.33% of the participants experienced abnormal glucose levels (hypoglycemia or hyperglycemia) between the checkpoint intervals of the intermittent A-line blood glucose measurements, even within the context of an intensive care unit.

In this prospective, proof-of-concept study, we showed that measurements taken by RTGMS have several advantages over the traditional intermittent A-line blood glucose monitoring. First, RTGMS is a less invasive method with a compact design that is suitable for use in infants. Second, the subcutaneous sensor performs well in the setting of sterile care with similar sensitivity and specificity compared with traditional intermittent A-line measurement. Finally, RTGMS sensors facilitate the identification and follow-up of the effects of interventions to better control blood glucose levels. In addition, RTGMS is capable of repeatedly measuring the duration, severity, and frequency of abnormal glucose in individual premature infants which will help physicians gain greater insights into dangerous blood glucose levels compared with A-line testing alone.

A-line catheterization is a common procedure within the neonatal intensive care unit, and it enables achieving continuous arterial blood pressure monitoring, arterial blood gases to monitor acid-base balance status and oxygenation, exchange transfusion, and frequent blood sampling. Compared with capillary blood glucose monitoring, A-line catheterization provides more reliable blood glucose values and avoids repeated skin puncturing [27].

However, traditional intermittent A-line blood glucose measurement does not totally meet the requirements of modern intensive care medicine, because hypoglycemia or hyperglycemia may influence neural and retinal development in premature infants [11, 28, 29]. Therefore, it is critical that the more accurate detectors are employed to monitor undiagnosed episodes of hypoglycemia or hyperglycemia. In addition, the repeated measurement of blood glucose levels using methods such as artery puncture or frequent heel sticks increases the risks of bruising, infection, and cartilage damage, particularly in premature infants [30]. RTGMS is an alternative means to continuously measuring blood glucose while simultaneously reducing the pain and disturbance associated with the traditional methods of repeated blood sampling.

Previous studies have comparing RTGMS with intermittent blood sampling in infant populations at risk of hyperglycemia and hypoglycemia have shown that RTGMS is comparable to [16] or exceeds [31] the detection capabilities of intermittent sampling. However, these studies have a number of limitations. One study had a small sample size of only 16 infants [16], and a larger study made only between-group comparisons owing to a shortage of direct RTGMS data [31], which could identify hyperglycemia and hypoglycemia within the checkpoint intervals of intermittent A-line sampling. To overcome these limitations, we evaluated a larger group of infants and directly compared RTGMS and intermittent A-line samples within the same individuals. However, the continuous glucose monitoring systems (CGMS) itself still presents with several limitations. For instance, biofouling may increase the risks of infection and inflammation. Moreover, ablation of host vasculature greatly affects the accuracy of blood glucose readings [32]. Essentially, the mechanism of CGMS contributes a 15–20 -min lag in the detection of interstitial blood glucose. This would reduce its reliability in blood glucose measurement processes in many circumstances, especially in hypoglycemic emergency situations and in situations that entail insulin adjustment [33]. Therefore, we used the Bland-Altman plot to evaluate the relative accuracy of all blood glucose values from CGMS, a shown in S1 Fig. Most of the readings were within the agreement intervals when CGMS was compared with the A-line measurement, and this frequency of correlation is still reasonable at a mean value of ≤40 mg/dL or ≥120 mg/dL. This indicates that the CGMS utilized in our study showed a certain degree of accuracy at extreme glucose levels.

Many studies have shown that the neonates born to diabetic mothers are at increased risk of blood glucose instability during the first days of life [13, 18, 19]. In this study, we further investigated whether the frequency of abnormal blood glucose levels in preterm babies with diabetic mothers is higher than premature infants with non-diabetic mothers. As shown in Fig 2, although there was no significant difference between the two groups, a trend wherein preterm infants with diabetic mothers exhibited higher instability of blood glucose levels was identified. Intensive A-line blood sampling every 6 hours may thus not be sufficient to detect abnormal glucose levels, particularly in high-risk infants during the first days.

The present study presents a standardized and tightly controlled comparison of intermittent and continuous blood glucose monitoring. However, our sample size was still not sufficiently large to comprehensively capture the effects of diabetic mothers on the glucose fluctuations of infants, especially for the blood glucose fluctuations of premature infants. However, we did identify a numerical, albeit nonsignificant, difference between diabetic and non-diabetic infants in terms of hyperglycemia/hypoglycemia alarm. Our sample size was sufficient to establish the reliability of RGTMS as a blood glucose measurement that is comparable, or arguably superior, to intermittent A-line sampling. A larger sample size would enable further investigation of the potential causal influence of diabetic mothers on infant blood glucose fluctuations. Apart from the limitation of sample size, our data were not corrected according to the sampled hematocrit data, and this could also result in possible bias.

To summarize, the present study demonstrated that RTGMS is comparable to A-line measurement for identifying blood glucose levels in premature infants. Moreover, RTGMS could identify substantial incidences of hypoglycemia and hyperglycemia that were undetected by A-line measurements. RTGMS is a less invasive method than traditional blood glucose measurement methods. It not only represents an efficient and effective method for identifying and tracking blood glucose abnormalities and infant responses to their associated treatments, but also can reduce the discomfort experienced in measurement processes by ensuring fewer blood test repeats and less handling to reduce the risk of infections and anemia [23–26].

## Supporting information

S1 Fig The Bland-Altman plot of the overall measuring of glucose level from PTGMS and A-Line. Glucose levels of preterm infants recorded by RTGMS and A-Line throughout the 72-hr were analyzed. To assess the degree of agreement of both method, several values were calculated. The overall sample size: 390; difference of mean: -0.2589; Stan Dev.: 12.5535; lower limits of agreement (LOA): -24.8638; upper LOA: 24.3459.
(PDF)
