# Supplementary material for: The value of real-time continuous glucose monitoring in premature infants of diabetic mothers
Source: PLoS One. 2017 Oct 16;12(10):e0186486. doi: 10.1371/journal.pone.0186486 (PMC5643124; doi:10.1371/journal.pone.0186486)
Supplement: S1 Fig — Glucose levels of preterm infants recorded by RTGMS and A-Line throughout the 72-hr were analyzed. To assess the degree of agreement of both method, several values were calculated. The overall sample size: 390; difference of mean: -0.2589; Stan Dev.: 12.5535; lower limits of agreement (LOA): -24.8638; upper LOA: 24.3459. (PDF) [file pone.0186486.s001.pdf]

Supporting information

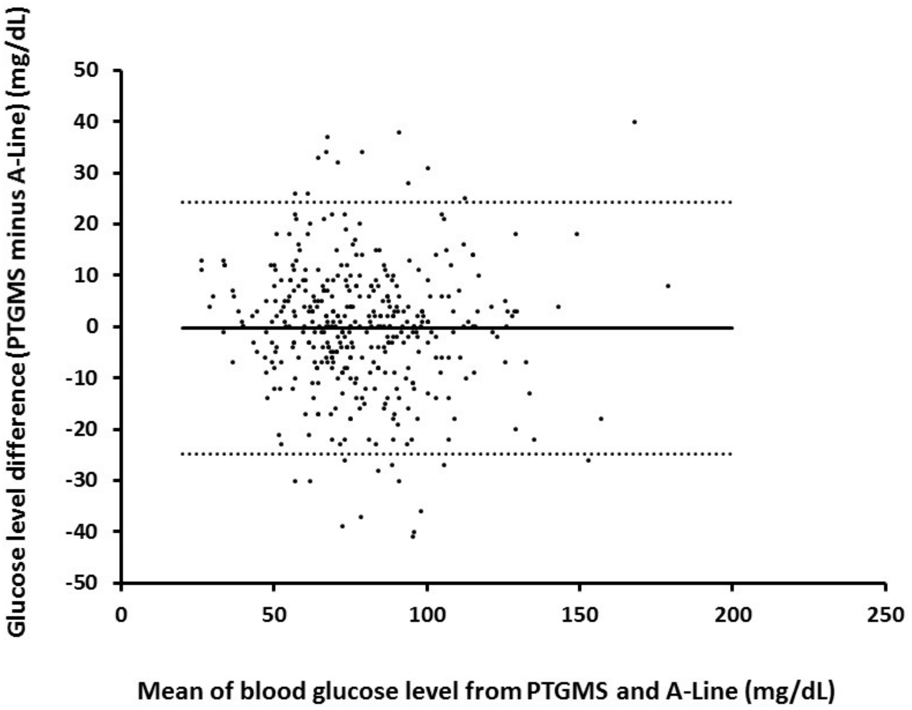

S1 Fig. The Bland-Altman plot of the overall measuring of glucose level from PTGMS and A-Line. Glucose levels of preterm infants recorded by RTGMS and A-Line throughout the 72-hr were analyzed. To assess the degree of agreement of both method, several values were calculated. The overall sample size: 390; difference of mean: -0.2589; Stan Dev.: 12.5535; lower limits of agreement (LOA): -24.8638; upper LOA: 24.3459.
